# Queries

**Published:** 1882-08

**Authors:** 


### QUERIES.

EDITOR REGISTER:—I would like some definite information concerning the use of lacto-phosphate of calcium. If some one who has used it will be kind enough to give us the benefit of their experience.

I wish to try its effects in two different cases; one in a young person at the age of puberty, with defective tooth structure; the other, a lady during gestation, with a view of its acting, more especially upon her own teeth than those of the child *in utero*.

I would like to know what proportion of the lacto-phosphate to use with a certain quantity of syrup for a dose; also, how often it should be taken, and how long continued to produce the desired effect.

The different authors which I have consulted are very indefinite; one gives the impression that it should be used a week at a time, for several months; Why not *constantly?* and if not, how often should it be repeated to be of permanent benefit?

